# T helper cell trafficking in autoimmune kidney diseases

**DOI:** 10.1007/s00441-020-03403-6

**Published:** 2021-02-17

**Authors:** Jan-Hendrik Riedel, Jan-Eric Turner, Ulf Panzer

**Affiliations:** 1 Division of Translational Immunology, III. Department of Medicine, University Medical Center Hamburg-Eppendorf, Martinistr. 52, 20246 Hamburg, Germany; 2 III. Department of Medicine, University Medical Center Hamburg-Eppendorf, Hamburg, Germany; 3 Hamburg Center for Translational Immunology, University Medical Center Hamburg-Eppendorf, Hamburg, Germany

**Keywords:** CD4, T cells, Crescentic glomerulonephritis

## Abstract

CD4^+^ T cells are key drivers of autoimmune diseases, including crescentic GN. Many effector mechanisms employed by T cells to mediate renal damage and repair, such as local cytokine production, depend on their presence at the site of inflammation. Therefore, the mechanisms regulating the renal CD4^+^ T cell infiltrate are of central importance. From a conceptual point of view, there are four distinct factors that can regulate the abundance of T cells in the kidney: (1) T cell infiltration, (2) T cell proliferation, (3) T cell death and (4) T cell retention/egress. While a substantial amount of data on the recruitment of T cells to the kidneys in crescentic GN have accumulated over the last decade, the roles of T cell proliferation and death in the kidney in crescentic GN is less well characterized. However, the findings from the data available so far do not indicate a major role of these processes. More importantly, the molecular mechanisms underlying both egress and retention of T cells from/in peripheral tissues, such as the kidney, are unknown. Here, we review the current knowledge of mechanisms and functions of T cell migration in renal autoimmune diseases with a special focus on chemokines and their receptors.

## Introduction

In addition to their critical role in defending against a wide array of invading microbes and pathogens, CD4^+^ T cells are key drivers of autoimmune diseases (Sallusto 2016). Their effector functions are mediated largely through the release of pro-inflammatory or regulatory cytokines. Based on their cytokine and transcription factor expression profile, CD4^+^ T cells can be classified into functionally distinct subsets, e.g., T_H_1, T_H_2, T_H_17, T follicular helper (T_FH_) cells and so far less well-characterized subtypes such as IL-9-producing T_H_9 cells and T_H_22 cells, as well as T regulatory (T_reg_) cells (O’Shea et al. 2010). Especially, T_H_1 and T_H_17 cells have been associated with the pathogenesis of autoimmune conditions, including crescentic glomerulonephritis, whereas Treg cells have been linked to the prevention of excessive immune responses in inflammatory diseases, including autoimmune renal diseases (Ghali et al. 2016; Kitching et al. 2000; Krebs et al. 2017).

Before CD4^+^ T cells can exert their local effects on renal damage or repair, they have to reach the site of inflammation. Migration of leukocytes into the kidney is a morphological hallmark of rapid progressive/crescentic glomerulonephritis. Infiltrating effector T cells may initiate and perpetuate glomerular and tubulointerstitial tissue injury, ultimately causing progressive loss of renal function (Suarez-Fueyo et al. 2017). It has become clear that chemokines and chemokine receptors are key regulators of directional T cell trafficking under homeostatic and inflammatory conditions (Griffith et al. 2014), which also applies to immune-mediated glomerular diseases (Chung et al. 2011). Interestingly, different CD4^+^ T cell subsets in humans and mice seem to display distinct patterns of chemokine receptor expression (Sallusto et al. 2000) that mediate their infiltration into the kidney but may simultaneously regulate T cell egress/retention.

## CD4^+^ T cell recruitment

**Fig. 1:**
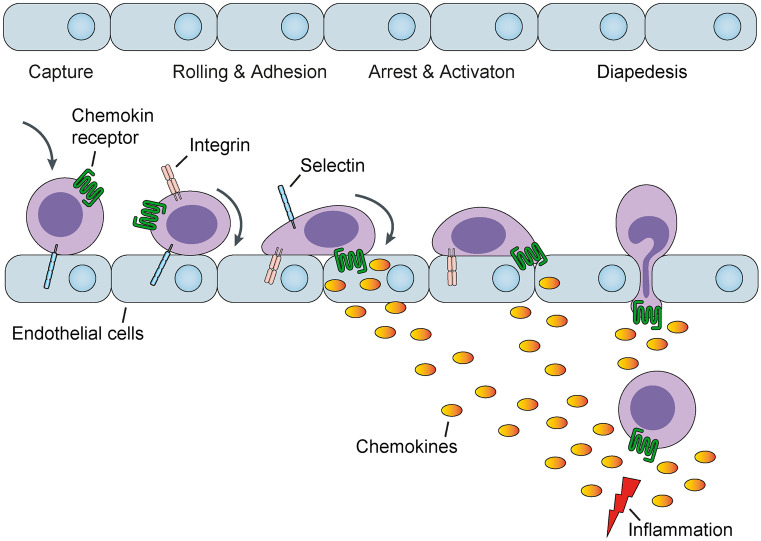
Overview of the processes leading to transmigration of leukocytes into inflamed renal tissue. Under inflammatory conditions, local renal cells increase the synthesis of chemokines and leukocytes residing inside the kidney capillaries are captured, engage in selectin-mediated rolling and are eventually arrested by interaction of integrins with the endothelial cell layer. The generated chemokines bind to endothelial cells through glycosaminoglycans thereby allowing their interaction with rolling and arrested leukocytes that bear their corresponding chemokine receptor leading to leukocyte activation. As a result of the massive chemokine production in areas of inflammation with subsequent diffusion to surrounding areas, a chemokine concentration gradient is formed between the site of tissue injury and vascular endothelium of neighboring blood vessels. Ultimately, the activated leukocytes adherent to the endothelial cell layer are thereby stimulated to leave the blood vessel, a process called diapedesis, being directed from areas of low to those of high chemokine concentrations, gradually reaching the tissue site of inflammation

The migration of CD4^+^ T cells from circulation to sites of tissue damage is a characteristic feature of almost every inflammatory process. Extravasation of T cells from the bloodstream into the tissue is a coordinated event that involves lymphocyte attachment to the vascular endothelium, followed by directional migration into the inflamed organ or tissue (von Andrian et al. 2000). This multifaceted process is controlled by several families of molecules, including selectins, integrins, chemokines and their respective receptors. Selectins mediate the primary contact between white blood cells and the blood vessel wall, leading to loose leukocyte rolling along the vascular endothelium. To prevent rolling leukocytes from being carried away by the blood flow, however, firm adhesion needs to be established. At this point, the molecule family of chemokines comes into play. At sites of tissue injury, a strong local production of chemokines is generally induced. Chemokines diffuse from the inflammatory epicenter into the surrounding tissue to adhere to and be presented by glycosaminoglycans of neighboring cell membranes (Panzer et al. 2006) (Fig. 1). Chemokines constitute a large family of small secreted proteins that play a unique role in directional migration and activation of T cells. On the basis of structural motifs near the N terminus, chemokines are separated into four distinct subfamilies (called C, CC, CXC and CX3C). T cells that are responsive to chemokines detect concentration gradients and migrate toward the source of chemokine secretion. T cell specificity is realized by a specific expression pattern of the corresponding chemokine receptors. T_H_1 cells express high levels of CXCR3 (Qin et al. 1998), CCR5 (Loetscher et al. 1998) and CXCR6 (Kim et al. 2001), whereas T_H_2 cells preferentially express CCR3 (Sallusto et al. 1997), CCR4 (Bonecchi et al. 1998) and CCR8 (Zingoni et al. 1998) and T_H_17 cells are positive for CCR6 (Acosta-Rodriguez et al. 2007; Hirota et al. 2007; Turner et al. 2010). In contrast, Tregs display a wider spectrum of chemokine receptors (Table 1).

**Table 1:** Role of chemokine receptor pathways for T cell trafficking and function in immune-mediated kidney diseases

T cell subsets	Chemokine receptor/main ligand(s)	Axis function/disease
T_H_1 cells	CCR5–CCL3-5 CXCR3–CXCL9-11	Deletion of CCR5 resulted in both pro- and anti-inflammatory responses in various GN models, most probably via upregulation of an alternative chemokine/chemokine receptor pathway (Anders et al. 2003; Panzer et al. 1999; Turner et al. 2012a, b; Turner et al. 2008) CXCR3 targeting resulted in impaired trafficking of pathogenic TH1 cells and an ameliorated course of crescentic and proliferative GN (Menke et al. 2008; Panzer et al. 2007; Steinmetz et al. 2009). In humans CXCR3^+^ T cells are recruited into inflamed kidneys, are enriched in urine and might be a biomarker of nephritis activity in SLE (Enghard et al. 2009)
T_H_17 cells	CCR6–CCL20	CCR6 is highly expressed on human and mouse T_H_17 cells. CCR6^+^ T_H_17 are enriched in the kidney of ANCA-GN patients (Krebs et al. 2016; Krebs et al. 2020). In experimental crescentic GN (NTN) CCR6^+^ T_H_17 are recruited via CCL20 into the inflamed kidney (Turner et al. 2010)
T_H_2 cells	CCR4–CCL17, CCL21 CCR8–CCL18	CCR4^+^ T lymphocytes in peripheral blood, which represent Th2 cells, preferentially migrate into the renal tissue of patients with lupus nephritis (Yamada et al. 2002) Targeting of CCL18/CCR8 had no major impact of T_H_2 response in experimental crescentic GN but reduced the infiltration of pathogenic mononuclear phagocyte and could serve as a biomarker for disease activity and renal relapse in ANCA-associated crescentic GN (Brix et al. 2015)
Tregs	CCR6–CCL20 CCR7–CCL19, CCL21 CXCR3–CXCL9-11	Stat3 activation leads to CCR6 expression on Tregs in mice and humans, which mediates specific control of T_H_17 immunity in several forms of experimental GNs (Kluger et al. 2014; Kluger et al. 2016; Turner et al. 2010) CCR7 deficiency exacerbates injury in crescentic GN due to aberrant localization of regulatory T cells (Eller et al. 2010) Tbet activation leads to CXCR3 expression on Tregs and mediates specific control of T_H_1 immunity (Nosko et al. 2017). Tregs expressing CXCR3 are enriched in the kidneys of patients with ANCA-associated crescentic GN and co-localize with CXCR3^+^ effector T cells (Paust et al. 2016)
T follicular helper (T_FH_) cells	CXCR5–CXCL15	CXCR5 is a marker for T_FH_ cells and promotes aberrant germinal center responses via IL-21 production with autoreactive memory B cell development and plasma cell-derived autoantibody production in SLE (Choi et al. 2017)
Natural killer T (NKT) cells	CXCR6–CXCL16	More than 90% of renal invariant NKT cells expressed CXCR6 and renal DCs produced high amounts of the cognate ligand CXCL16 in GN, suggesting that renal DC-derived CXCL16 might attract protective CXCR6^+^ invariant NKT cells (Riedel et al. 2012)

## T_H_1 cell trafficking in renal inflammation

**Fig. 2:**
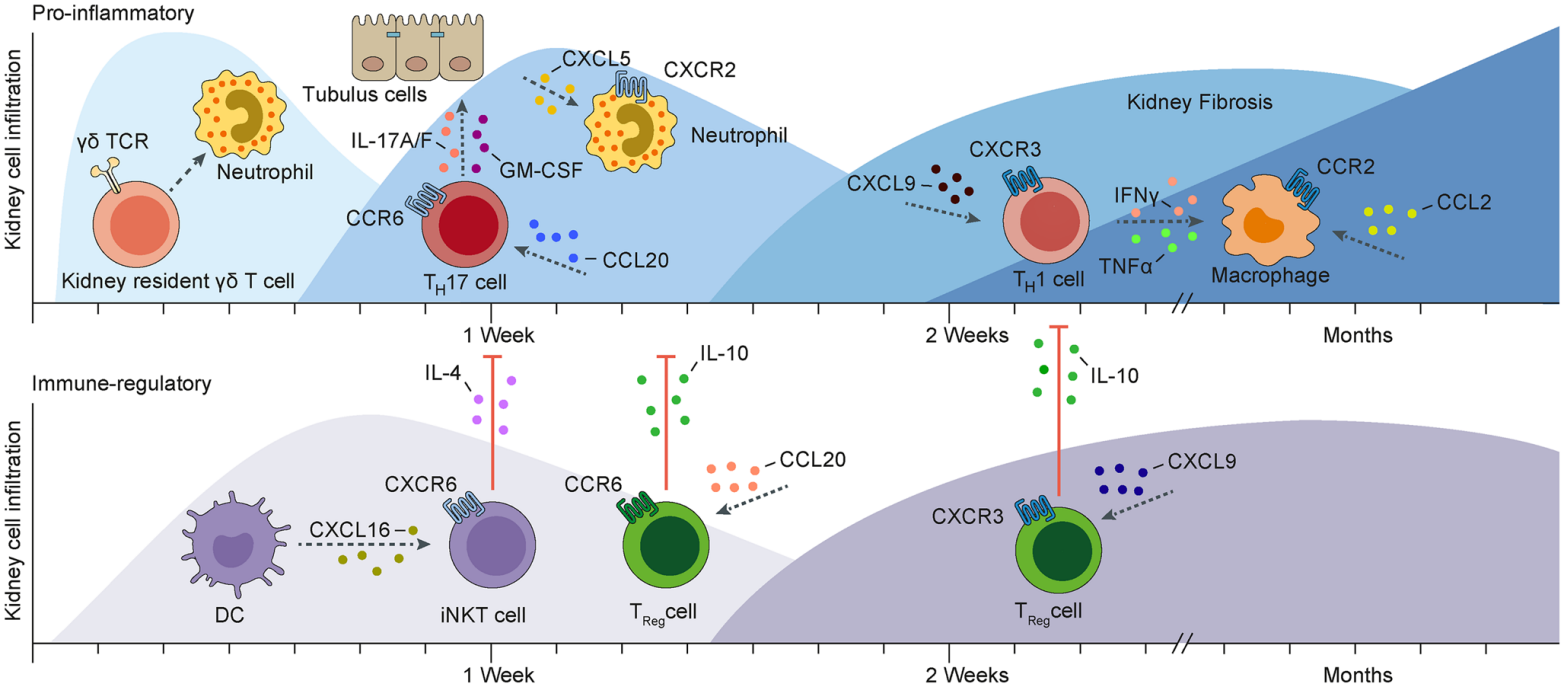
The time-dependent changes of pro-inflammatory and immune-regulatory functions of leukocyte subsets during the course of experimental crescentic glomerulonephritis (NTN) are shown. NTN is induced by an injection of a heterologous sheep-anti-mouse serum and, within a few hours, resident γδ T cells activate neutrophils. Shortly after, the autologous pro-inflammatory immune response is firstly mediated by the recruitment of CCR6 expressing T helper 17 (T_H_17) cells in response to local CC-chemokine ligand (CCL)20 production. These infiltrating T_H_17 cells produce pro-inflammatory cytokines, i.e., interleukin (IL)-17A, IL-17F and GM-CSF, leading to the recruitment and activation of tissue disruptive neutrophils. Later, the recruitment of CXCR3 expressing T helper 1 (T_H_1) cells in response to local CXCL9 production prevails. These infiltrating T_H_1 cells produce cytokines such as interferon-γ (IFNγ) and tumor necrosis factor α (TNFα), which are potent activators of macrophages, leading to their releasing injurious mediators such as nitric oxide. Simultaneously, during the first days of NTN, dendritic cells (DCs) attenuate crescentic glomerulonephritis by attracting regulatory invariant natural killer T (iNKT) cells via the CXC-chemokine ligand (CXCL) 16–CXCR6 axis and these cells produce IL-4 thereby reducing destructive T_H_17 cell responses. At a later stage, CCR6^+^ and CXCR3^+^ regulatory T (T_reg_) cells are recruited into the inflamed kidney, respectively and protect against an overwhelming T_H_17 cell- and T_H_1 cell-mediated immune response, at least partly through the local production of IL-10. CCR, CC-chemokine receptor; CXCR, CXC-chemokine receptor; TCR, T cell receptor; GM-CSF, granulocyte–macrophage colony-stimulating factor

The introduction of the pathogenic function of T_H_1 cells into the field of renal autoimmune disease by the Holdsworth, Tipping, and Kitching group (Kitching et al. 2000) provided a rationale for studying the impact of “T_H_1 chemokine receptors” CXCR3, CCR5 and CXCR6 for trafficking of T_H_1 cells to the kidney. It was shown that CXCR3-deficient mice were partly protected from immune-mediated injury in both the nephrotoxic nephritis (NTN) model of crescentic glomerulonephritis and the MRL-*Fas*^lpr^ model of murine lupus nephritis (Menke et al. 2008; Panzer et al. 2007; Steinmetz et al. 2009). This effect was most likely attributable to the impaired trafficking of pathogenic T_H_1 cells. In human renal inflammatory disease, we and other groups found infiltrating CXCR3^+^ T cells in the periglomerular area and tubulointerstitium (Panzer et al. 2004; Segerer et al. 2004), which correlated with an unfavorable clinical outcome. Moreover, urinary mRNA levels of the CXCR3 ligand CXCL10 and CXCR3^+^ CD4^+^ T cells in the urine were suggested as non-invasive tools for monitoring the activity in lupus nephritis and might therefore provide a new biomarker for acute nephritis flares in systemic lupus erythematosus patients (Enghard et al. 2009). The role of CCR5 and CXCR6, expressed on T_H_1 cells, seems to be more complicated. Targeting of CCR5 or its ligand CCL5 resulted in both pro- and anti-inflammatory responses in various models of glomerulonephritis, most probably via upregulation of an alternative chemokine/chemokine receptor pathway (Anders et al. 2003; Panzer et al. 1999; Turner et al. 2012a, b; Turner et al. 2008). Finally, using CXCR6-deficient mice, we unexpectedly identified the importance of the CXCR6/CXCL16 axis for the recruitment and activation of protective iNKT cells in nephrotoxic nephritis without affecting T_H_1 cell trafficking (Riedel et al. 2012) (Fig. 2).

## T_H_17 cell trafficking and function in immune-mediated glomerular diseases

The critical role of IL-17A and T_H_17 cells in experimental crescentic glomerulonephritis was first shown in experimental models of glomerulonephritis, using mice with IL-23 p19, IL-17A, IL-17F, IL-17C and RORγt gene deficiency that all show impaired T_H_17 immune responses. These knockout mice, as compared to their wild-type littermates, were much less susceptible to experimental models of crescentic glomerulonephritis (Kluger et al. 2014; Krohn et al. 2018; Kyttaris et al. 2010; Ooi et al. 2009; Paust et al. 2009; Riedel et al. 2016; Steinmetz et al. 2011; Tulone et al. 2011). T_H_17 cells highly express CCR6 and are recruited via local CCL20 formation into the kidney (Turner et al. 2010). Of note, during early stages of experimental GN, γδ T cells are the major cellular source of IL-17A in the kidney but at later stages CD4^+^ T_H_17 cell-derived IL-17A drives CXCL5 expression in kidney tubular cells, leading to recruitment of CXCR2^+^ neutrophils that contribute to renal tissue injury (Disteldorf et al. 2015; Turner et al. 2012a, b). Furthermore, it was shown that CD4^+^ T cell-derived IL-17F drives renal tissue injury in a non-redundant function in acute crescentic GN and in the chronic model of pristane induced systemic lupus by induction of the chemokines CXCL1 and CXCL5 in resident kidney cells, again by recruiting tissue destructive neutrophils (Riedel et al. 2016) (Fig. 2).

In addition, we reported that, compared with healthy controls, ANCA-GN patients had significantly elevated serum levels of IL-17C. In mouse models of crescentic GN (nephrotoxic nephritis) and pristane-induced lupus nephritis, deficiency in IL-17C significantly ameliorated the course of GN in terms of renal tissue injury and kidney function. Deficiency of the unique IL-17C receptor IL-17 receptor E (IL-17RE) provided similar protection against crescentic GN. These protective effects associated with a reduced T_H_17 response. IL-17RE was highly expressed by T_H_17 cells and loss of this expression prevented the T_H_17 response and subsequent tissue injury in crescentic GN. These findings suggest that IL-17C promotes T_H_17 cell responses and immune-mediated kidney disease via IL-17RE expressed on T_H_17 cells (Krohn et al. 2018). These findings are of great interest, because anti-T_H_17/IL-17 treatment has been approved for the treatment of psoriasis and might also represent an attractive therapeutic strategy in T-cell-driven GN. This concept is further supported by the identification of high frequencies of CCR6 + RORγt + T_H_17 cells in kidney biopsy samples from patients with ANCA-associated glomerulonephritis. Of note, T_H_17 cells were barely detectable in the peripheral blood of these patients, suggesting specific recruitment and accumulation of T_H_17 cells into the kidney (Krebs et al. 2016).

## Role of Treg cell trafficking in GN

In the last few years, anti-inflammatory properties of regulatory T cells in experimental GN have been demonstrated by genetic or antibody-mediated ablation of Treg cells and adoptive Treg transfer experiments (Ooi et al. 2011; Paust et al. 2011; Wolf et al. 2005). In nephrotoxic nephritis, CCR7 was crucial for guiding Tregs to lymph node areas, where they attenuated disease by suppressing effector T cell activation (Eller et al. 2010) and the S1PR1-modulator FTY720 (Fingolimod) induced Treg trapping in draining lymph nodes under inflammatory conditions while simultaneously reducing their suppressive capacities through inhibition of Treg expansion (Wolf et al. 2009). We were able to show that Tregs also infiltrate the inflamed kidney in a CCR6-dependent manner and suppress the intrarenal immune effector phase (Turner et al. 2010) (Fig. 2). It is, however, still unclear whether additional chemokine receptors (or receptor combinations) are crucial for Treg cell localization and function. Flow cytometry-based analysis of the chemokine receptor expression profile of Tregs in renal biopsies from patients with ANCA-associated GN revealed expression of CCR4, CCR6 and CXCR3 on these cells (Paust et al. 2016). In particular, Tregs expressing the T_H_1-associated chemokine receptor CXCR3 were enriched in the kidneys of patients with ANCA-GN, as compared to Tregs from peripheral blood and from the tonsil. Furthermore, these CXCR3^+^ Tregs co-localized with CXCR3^+^ effector T cells. Therefore, a specific recruitment process of CXCR3^+^ Tregs to the kidney in ANCA-GN can be assumed. Treg-specific deletion of CXCR3 resulted in reduced Treg infiltration and in a selective up-regulation of pathogenic T_H_1 cell responses in the kidney after induction of experimental glomerulonephritis (NTN model) (Paust et al. 2016). As a consequence of the excessive T_H_1/IFN-γ response, an aggravated course of renal disease was observed*.* These findings indicate that control of T_H_1-type inflammation in autoimmunity and infection requires a “customized” counterbalance that critically depends on CXCR3^+^ Tregs with trafficking properties of T_H_1 cells (Koch et al. 2009; Nosko et al. 2017). These findings will help to better understand the specificity, stability and functional activity of Treg cells and finally, to encourage the clinical use of adoptive Treg cell therapy in immune-mediated diseases (Raffin et al. 2020). Properties and functions of Tregs are described in more detail in another review article of this special issue of *Cell & Tissue Research* by Herrnstadt and Steinmetz.

## Renal CD4^+^ T cell emigration

T cell exit from inflamed tissues via lymphatic vessels to the draining lymph node might represent a previously unrecognized mechanism for regulation of the renal T cell infiltrate and might thereby play a hitherto underestimated role in the resolution of inflammation. The mammalian kidney has a rich supply of lymphatic vessels that mainly follow the routes of the renal vasculature, most abundant in the interstitium around interlobular, arcuate and interlobar blood vessels (Ishikawa et al. 2006). Lymphatic vessels are composed of partly overlapping lymphatic endothelial cells (LECs) that interconnected via discontinuously arranged junctions, removing fluid and macromolecules from the interstitial space (Russell et al. 2019). Under inflammatory conditions macromolecules (e.g., antigens) and leukocytes (e.g., dendritic cells (DCs) and T cells) may reach the renal draining lymph node via afferent lymphatic vessels thereby modulating the local inflammatory milieu (Itano et al. 2003; Mackay et al. 1990). In addition, lymph vessels are able to be newly formed in a term called neolymphangiogenesis. In the kidney, this mainly occurs in conditions associated with inflammation and/or fibrosis and in kidney transplantation (Kerjaschki et al. 2004; Seeger et al. 2012; Stuht et al. 2007; Yazdani et al. 2014) but because data on the functional role of neolymphangiogenesis in kidney disease remain scarce, its correlative or causative nature continues to be speculative. Mainly due to the lack of suitable experimental systems to track egress of cells from non-lymphoid tissue, e.g., the kidney, to the draining lymph node, the mechanisms of T cell tissue exit still remain largely uninvestigated (Permanyer et al. 2018). Since signals that drive T cell egress and those that authorize T cell retention in peripheral tissues are presumably closely connected, these questions are also central in understanding the biology of tissue-resident memory T cells (T_RM_) (Szabo et al. 2019). Indeed, the kidney might prove to be an ideal location to study the functional role of T cell egress, because there is limited afferent lymphatic drainage with only a single direct draining lymph node (Jafree et al. 2020). Removal of the kidney draining lymph nodes before the initiation of experimental GN (NTN) led to the amelioration of kidney injury with lower numbers of T cells and macrophages in the kidney (Kasinath et al. 2019). Since the lymph nodes were removed before disease initiation, we cannot deduce effects of the removal on T cell egress in the effector phase of disease. The distinctive anatomy of the renal lymphatic drainage system was also used in a set of studies by the Bromberg group, analyzing the emigration properties of Tregs transplanted with islet grafts underneath the renal capsule (Zhang et al. 2004). They showed that these Tregs use diverse chemokine receptors and the sphingosine 1-phosphate receptor (S1PR)1 to emigrate from the kidney tissue via afferent lymphatics to the kidney draining lymph node (Xiong et al. 2016; Zhang et al. 2009). The artificial nature of the aforementioned studies together with the fact that knowledge about effector T cell egress from other organs, namely skin (Brown et al. 2010; Debes et al. 2005; Geherin et al. 2014) and lung (Bromley et al. 2005; Caucheteux et al. 2013), is exclusively derived from adoptive T cell transfer models, leads to extreme caution in drawing conclusions about T cell egress in autoimmune/inflammatory (kidney) diseases. Hence, the potential role of T_H_1 and T_H_17 cell egress in regulating the inflammatory infiltrate in murine and human autoimmune kidney diseases remains to be elucidated.

In our own preliminary experiments, treatment with the S1PR1-modulator FTY720 (Fingolimod), reported to block the exit of T cells from lymph nodes, in crescentic GN (NTN) caused enhanced accumulation of T cells in the kidney, in line with data from the lung (Cose et al. 2006; Sawicka et al. 2003) and the skin (Brown et al. 2010), accompanied by an aggravated course of GN, potentially as a consequence of inhibition of renal T cell exit (unpublished data). These findings suggest a biological role of T cell exit in renal inflammation. An established experimental system for labeling intrarenal lymphocytes, using transgenic mice ubiquitously expressing the photoconvertible fluorescent protein Kaede, will allow studying mechanisms of renal T cell egress, e.g., to the renal lymph node (Krebs et al. 2016; Tomura et al. 2010). By combining this method with immunohistochemical analysis of renal biopsies from patients with crescentic and proliferative GN, we showed constitutive upregulation of the chemokine CCL21 in periglomerular lymphatic vessels and CCR7-dependent emigration of dendritic cells from the kidney to the renal lymph node under homeostatic and nephritic conditions (*manuscript in preparation*). While these findings are in line with published results from the skin (Brown et al. 2010; Gomez et al. 2015), we found that, in contrast to skin T cells, CD4^+^ T cells in the inflamed kidney do not rely on CCR7 for kidney egress, indicating that other chemokine receptors might be crucial in this process. We hypothesize that under inflammatory conditions lymphatic endothelial cells in the kidney might express alternative chemokines (in addition to CCL21). This could stimulate leukocytes to exit the kidney via afferent lymphatics to migrate to the draining lymph node and, potentially, to recirculate throughout the body.

Additional experiments using Kaede mice with the respective chemokine receptor knockouts to assess the functional involvement of selected chemokine receptors (e.g., CXCR3 and CCR6) in the egress of T cell subsets from the kidney under nephritic conditions might be of great interest for a better understanding of this process. The Kaede technique will also help to characterize the phenotype and chemokine receptor profiles of emigrated cells in the kidney draining lymph node, using FACS- and scRNA-seq-based analyses and compare them with that of non-emigrating leukocytes. Flow cytometry and scRNA-seq analyses will allow “interactom” studies of murine and human renal T cells and kidney lymphatic endothelial cells by computational modeling algorithms aimed at analyzing the potential communication between cells via ligand and receptor interactions. Taken together, these recent technical advances will enable a comprehensive understanding of (CD4^+^) T cell emigration in health and disease.

## Role of T helper cell proliferation and cell death in renal inflammation

Many of the effects of T cells on renal damage and repair depend on their presence at the site of inflammation. There are two other major factors besides T cell trafficking that could regulate the T cells infiltrate in the kidney in principal, i.e., T cell proliferation and T cell death but their roles in crescentic GN have hardly ever been characterized. While it is known that after intrarenal antigen-placement, antigen-specific T cells proliferate in the draining renal lymph node under inflammatory conditions and later accumulate in the kidneys (Dong et al. 2005; Edgtton et al. 2008), direct evidence for (a functional role) of intrarenal T helper cell proliferation is largely missing. Recently, a study supplied evidence for a protective effect of treatment with the glucagon-like peptide-1 (GLP1) receptor agonist liraglutide in murine crescentic GN (Moschovaki Filippidou et al. 2020). Among other mechanisms, the proliferation of renal cells in glomeruli as well as within the periglomerular region was significantly reduced and ex vivo treatment of polarized T_H_1 and T_H_17 cells with liraglutide likewise significantly decreased their proliferation. Cell death (e.g., apoptosis, necroptosis, ferroptosis and pyroptosis) occurs on a considerable scale both as a consequence of inflammation and as a trigger of inflammation in various forms of kidney disease (Sarhan et al. 2018). However, available studies mainly referred to resident kidney cells and T cell death has mainly been studied in the context of analyses of the systemic immune response in models of crescentic glomerulonephritis, for example in the analysis of nephritic *TBX21* deficient mice (Phoon et al. 2008). Although kidney infiltrating CD4^+^ T cells express significant amounts of programed cell death-1 (PD-1) and/or programed cell death ligand-1 (PD-L1), analyses of these molecules showed major roles in regulating proliferative/suppressive capacities and changing cytokine production without affecting cell death itself (Kasagi et al. 2010; Neumann et al. 2019). A recent publication showed that in hypoxic renal tissue as a consequence of inflammation in lupus-prone mice, renal infiltrating CD4 and CD8 T cells in fact become more resistant to cell death by the expression of hypoxia-inducible factor-1 (HIF-1) and that HIF-1-dependent gene-regulated pathways were also upregulated in renal-infiltrating T cells in human lupus nephritis (Chen et al. 2020). This argues against a physiological role of T cell death as a means of reducing the inflammatory renal T cell infiltrate in kidney disease.

## Origin of renal T_H_17 cells

**Fig. 3:**
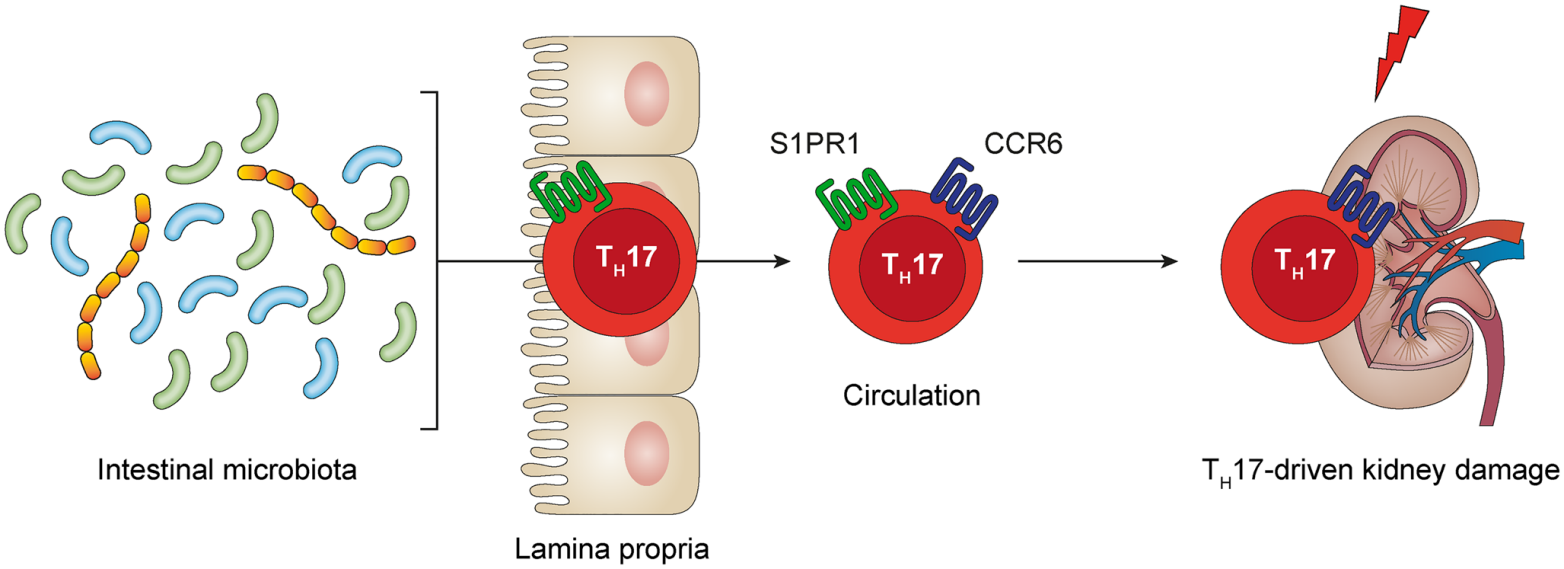
Intestinal microbiota-induced T_H_17 cells egress from the gut dependent on S1PR1 into the circulation, in this way serving as an intestinal T_H_17 cell “reservoir” and infiltrate the kidney via  chemokine receptor CCR6 to mediate kidney damage in crescentic glomerulonephritis. CCR, CC-chemokine receptor; S1PR, sphingosine 1-phosphate receptor

It has become clear that T_H_17 cells are predominantly found in the small intestinal lamina propria under homeostatic conditions where they are induced by specific adhesive microorganisms (Atarashi et al. 2015; Ivanov et al. 2009; Sano et al. 2015). Antigens expressed by these adhesive microorganisms, such as segmented filamentous bacteria (SFB), are presented in the mesenteric lymph nodes to naïve CD4^+^ T cells. After activation and differentiation, these T cells migrate into the intestinal lamina propria and produce IL-17A upon antigen recognition. This concept is supported by the abundance of SFB-specific T_H_17 cells in the small intestinal wall (Ivanov et al. 2009, 2008; Yang et al. 2014). Moreover, germ-free mice (GFM) that are devoid of bacterial colonization and antibiotic-treated mice with altered intestinal bacteria have reduced numbers of T_H_17 cells in the intestine (Ivanov et al. 2009, 2008), leading to abrogated extraintestinal T_H_17 immune responses in models of EAE and arthritis (Lee et al. 2011; Wu et al. 2010). Using Kaede-transgenic mice, which ubiquitously express photoconvertible Kaede protein, we demonstrated that after the induction of experimental crescentic glomerulonephritis (cGN), intestinal T_H_17 cells migrate into the inflamed kidney (Krebs et al. 2016). In this process, emigration of CD4^+^ T cells from the small intestine depended on S1PR1 and infiltration of intestine-derived CCR6-expressing T_H_17 cells into the kidney was mediated by chemokine CCL20. The relationship between intestinal microbiota and T_H_17 cells in the kidney is of functional importance as GFM were protected from T_H_17-mediated tissue damage in experimental cGN (Krebs et al. 2016). Even more important, therapeutic invention using the orally administered antibiotic vancomycin led to a reduction in renal T_H_17 cells and reduced renal tissue damage. Together, these findings suggest that the intestine serves as a reservoir from which T_H_17 cells can be recruited to specific extraintestinal sites in autoimmune disease (Fig. 3). A potential link between intestinal and renal T_H_17 cells in human GN might render these cells susceptible to manipulations of the intestinal microbiome. Since T_H_17 cells are abundant in the kidney of patients with ANCA-associated crescentic glomerulonephritis (Couser 2012; Krebs et al. 2016, 2020), this axis might be of high relevance in the development of new T_H_17-directed therapeutic strategies.

## Trafficking of other T cell subsets

Knowledge about a role of other CD4^+^ T cell subsets, i.e., T_H_9 (Eller et al. 2011; Xiong et al. 2020), T_H_22 (Gnirck et al. 2019) and T_FH_ cells (Liarski et al. 2014), in human or experimental crescentic GN is still sparse and even less is known about their specific trafficking properties in inflammatory kidney disease. For IL-4- and IL-10-producing immunomodulatory iNKT cells, a dependency on the chemokine receptor CXCR6 for recruitment into the inflamed kidney has been shown (Riedel et al. 2012). The chemokine receptor’s cognate ligand CXCL16 is produced by immature dendritic cells (DCs) in the early phase of experimental crescentic GN to recruit iNKT cells to the kidney. Properties and functions of γδ T cells, tissue resident memory T cells and CD8^+^ T cells are described in detail in further review articles of this special issue of *Cell & Tissue Research* by Mittrücker et al. and Neumann et al., respectively.

## Targeting T cell trafficking in human autoimmune diseases

During the last few years, evidence has been accumulating that the circulating T cell pool contains multiple antigen-experienced subsets bearing distinct tissue tropisms. For example, the active form of vitamin D3 instructs T cells to express CCR10, enabling them to migrate towards the skin-specific chemokine CCL27 secreted by keratinocytes of the epidermis (Sigmundsdottir et al. 2007). In another landmark study, it was shown that Peyer’s patch dendritic cells imprint gut-homing specificity on T cells by inducing their expression of the integrin α4β7 and the chemokine receptor CCR9, the receptor for the gut-associated chemokine CCL25, thus licensing T cells to access anatomical sites most likely to contain their cognate antigen (Mora et al. 2003). Based on these findings the monoclonal antibody Vedolizumab, binding the integrin α4β7 on the surface of leukocytes hindering their infiltrating into gut tissue, has been in use for several years in inflammatory bowel disease (Feagan et al. 2013; Sandborn et al. 2013). Antagonists of CCR9 have also been developed, so far showing conflicting results in inflammatory bowel disease (Bekker et al. 2015; Feagan et al. 2015). In multiple sclerosis (MS), entrance of leukocytes into the central nervous system (CNS) can be reduced by sequestering pathogenic leukocytes in the lymph nodes by application of fingolimod, a sphingosine-1-phosphate receptor modulator and by hampering their passing through the blood–brain barrier, by blocking the integrin α4β1 on leukocytes with the monoclonal antibody natalizumab (Polman et al. 2006). These proof of principle studies provided evidence that targeting T cell trafficking in human autoimmune diseases is feasible and represents an attractive therapeutic approach.

## Concluding remarks

Although recruitment of T cells to the kidney in a wide array of autoimmune kidney diseases has been observed for almost two decades and constitutes a hallmark of rapid progressive crescentic glomerulonephritis (Couser 2012; Kurts et al. 2013), no therapeutic tool specifically targeting leukocyte trafficking has been introduced into the armamentarium of nephrologists so far. New technical developments, such as single-cell RNAseq analysis, will allow for a detailed characterization of the expression pattern of a wide array of trafficking receptors in blood samples and renal biopsies of patients with glomerulonephritis as well as in samples from various murine tissues, i.e., blood, kidney and lymphatic organs, in experimental crescentic glomerulonephritis. This analysis might help to identify and characterize a specific T cell migration post code for the kidney, such as CCR10 for the skin and CCR9 for the intestine. Potential molecular mediators involved in the pathophysiology of kidney disease and their functional relevance may then be validated in preclinical disease models and epidemiological studies before potentially being transferred to clinical trials. In summary, future studies will result in a better understanding of chemokine/chemokine receptor-regulated T cell trafficking and its role in renal tissue injury. This might facilitate target-specific therapies in human T cell-mediated glomerulonephritis.
